# Knockdown of LMX1B Suppressed Cell Apoptosis and Inflammatory Response in IL-1*β*-Induced Human Osteoarthritis Chondrocytes through NF-*κ*B and NLRP3 Signal Pathway

**DOI:** 10.1155/2022/1870579

**Published:** 2022-09-12

**Authors:** Yiping Mu, Lining Wang, Ling Fu, Qi Li

**Affiliations:** ^1^Hand Surgery Department, Central Hospital Affiliated to Shenyang Medical Collage, Shenyang Clinical Research for Hand and Foot Diseases, Shenyang, 110024 Liaoning Province, China; ^2^Laboratory Department, Central Hospital Affiliated to Shenyang Medical College, Shenyang, 110024 Liaoning Province, China

## Abstract

Osteoarthritis (OA), a chronic degenerative joint disease, always occurred in the aging population. There is evidence suggests that chondrocytes' survival, inflammation, and apoptosis play critical roles in OA pathogenesis. LMX1B has been shown to be involved in antiosteogenic function in early patterning of the calvaria. However, the role and mechanism of LMX1B in OA is not unknown. The present study observed that LMX1B was highly expressed in OA patients compared with normal patients. Besides, we found that IL-1*β* increased LMX1B mRNA and protein expression in SW1353 and C28/I2 chondrocytes. LMX1B knockdown increased IL-1*β*-induced cell viability and proliferation and suppressed cell apoptosis and inflammation response, including IFN-*γ*, TNF-*α*, IL-6, prostaglandin E2 (PGE2), and NO both in SW1353 and C28/I2. Furthermore, LMX1B silence inhibited MMP-3 and MMP-13 expression both in SW1353 and C28/I2 cells. Also, the activation of the NF-*κ*B and NLRP3 signaling pathway was suppressed in LMX1B silence cells by decreasing the p-p65 and NLRP3 protein expressions. Additionally, inhibition of NF-*κ*B by PDTC suppressed NLRP3 expression. Moreover, NLRP3 overexpression reversed the effects of LMX1B silence on chondrocytes' survival, proliferation, apoptosis, and inflammation. Finally, we confirmed that LMX1B depletion had protective effects in OA rats *in vivo*.

## 1. Introduction

Osteoarthritis (OA) is an age-related disease that affects axial and peripheral articulations and weight-bearing joints affecting tens of millions of people all over the world [1]. The disorder is characterized by inflammation of synovial joints, anabolism of articular cartilages, degradation of articular cartilage, improper catabolism, and thickening of subchondral bone [2, 3]. In the progression of OA, inflammatory cytokines like IL-1*β* resulted in tissue injury and articular cartilage degeneration of the OA joints and associated with many pathological processes, including metabolic imbalance, hypertrophy, apoptosis of chondrocytes, and dysregulation of autophagy [4–7]. Although some therapeutic approaches have been used for OA, such as surgical replacement of joint and pain management, these treatments were not satisfactory, and the loss of articular cartilage could not be reversed [8, 9]. Therefore, it will be necessary to find and understand the effective molecular and associated mechanisms that contributed to the pathological processes of chondrocytes inflammation and apoptosis for OA treatment.

LIM homeobox transcription factor 1 beta (LMX1B) which consists of 395 amino acid residues was located on autosomal 9q34.1 and encodes by *LMX1B* gene that contains eight exons [10]. LMX1B is a transcription factor and belongs to LIM homeodomain family proteins [10, 11]. It is reported that LMX1B was involved in organ and extremity development, including limb, kidney, brain, eye, glomerular basement membrane, and neuron; moreover, LMX1B has been shown implicated in cancer development [11–16]. Moreover, emerging studies have revealed that LMX1B plays a vital function in the development of the central nervous system, limbs, and kidney [15, 17, 18]. Also, evidence suggested that LMX1B plays important roles in the cell apoptosis inhibition under hypoxia and reoxygenation condition in renal tubular epithelial cells [19]. However, the specific role and molecular mechanism of LMX1B in IL-1*β*-induced human osteoarthritis chondrocytes are poorly understood.

Nod-like receptor protein 3 (NLRP3) inflammasome, known as innate immune sensors, contains NLRP3 protein, pro-caspase-1, and apoptosis-associated speckle-like protein which is involved in cell apoptosis and inflammation response [20, 21]. There is convincing evidence that the elevation of NLRP3 is closely connected with proinflammatory cytokine production, including tumor necrosis factor-*α* (TNF-*α*), interleukin-6 (IL-6), and interleukin-1*β* (IL-1*β*) [22]. Moreover, the nuclear factor-kappaB (NF-*κ*B) transcription factor is a major mediator of immune homeostasis and inflammation, and evidence suggested that NF-*κ*B plays a central role in the inflammation response [23]. However, whether LMX1B regulated NF-*κ*B and NLRP3 signal pathway and involved in OA development remains unclear. In the current study, we determined the specific role of LMX1B on cell apoptosis and inflammatory response in IL-1*β*-induced human osteoarthritis chondrocytes. Our study uncovers the role of LMX1B and its regulation of NF-*κ*B and NLRP3 signal pathway in chondrocytes.

## 2. Methods

### 2.1. Patients

Tissue collection in this study was approved by the Ethic Committee of Central Hospital Affiliated to Shenyang Medical Collage (Shenyang) and according to the principles of the Declaration of Helsinki. Human cartilage samples were obtained from 20 osteoarthritis patients (age 58.2 ± 9.8) undergoing total knee arthroplasty and 10 healthy donors (age 55.4 ± 7.6) undergoing total hip replacement surgery because of femoral neck fracture. Moreover, healthy donors do not have arthritis or rheumatoid arthritis. The informed consent form was obtained from all donors. Tissue samples were collected after surgery and stored at -80°C.

### 2.2. Cell Culture and Treatment

Human chondrosarcoma cell line SW1353 and C28/I2 cells (American Type Culture Collection, Manassas, VA, USA) were maintained in Dulbecco's modified Eagle medium (DMEM), supplemented with 10% fetal bovine serum in humidified incubated air with 5% CO_2_ at 37°C. IL-1*β* was purchased from the R&D Systems (Abingdon, UK) and dissolved in phosphate-buffered saline (PBS). SW1353 and C28/I2 cells were treated with 10 ng/ml IL-1*β* to simulate osteoarthritis injury.

### 2.3. Cell Grouping and Transfection

The SW1353 cells were divided into seven groups: control group, IL-1*β* group, IL-1*β*+control siRNA group, IL-1*β*+LMX1B siRNA group, IL-1*β*+PDTC group, IL-1*β*+LMX1B siRNA+pcDNA3.1 group, and IL-1*β*+LMX1B siRNA+pcDNA3.1-NLRP3 group. The C28/I2 cells were divided into four groups: control group, IL-1*β* group, IL-1*β*+control siRNA group, and IL-1*β*+LMX1B siRNA group. In the IL-1*β* group, SW1353 and C28/I2 cells were treated with 10 ng/ml IL-1*β* for 24 h. In the IL-1*β*+control siRNA group and IL-1*β*+LMX1B siRNA group, the SW1353 and C28/I2 cells were transfected with control siRNA or LMX1B siRNA for 24 h by using Lipofectamine 2000 reagent and then treated with 10 ng/ml IL-1*β* for 24 h. In the IL-1*β*+PDTC group, SW1353 cells were treated with 10 ng/ml IL-1*β* and PDTC for 24 h. In the IL-1*β*+LMX1B siRNA+pcDNA3.1 group and IL-1*β*+LMX1B siRNA+pcDNA3.1-NLRP3 group, SW1353 cells were cotransfected with LMX1B siRNA and pcDNA3.1 control vector or LMX1B siRNA and pcDNA3.1-NLRP3 vector for 24 h and then treated with 10 ng/ml IL-1*β* for 24 h.

### 2.4. Cell Viability Assay

SW1353 and C28/I2 cell viability was determined using a Cell Counting Kit-8 (CCK8) assay. Briefly, SW1353 cells were seeded in 96-well plates at a density of 5 × 10^3^ cells/well for 24 h and then treated and transfected as above. At the appropriate time, CCK8 solution (10 *μ*l) was added to each well then cultured at 37°C for another 4 h. Absorbance at a wavelength of 490 nm was measured and recorded with a microplate reader (Model 680; Bio-Rad, Hercules, CA, USA).

### 2.5. Cell Proliferation Assay

SW1353 and C28/I2 cell proliferation was determined using 5-ethynyl-2′-deoxyuridine (EdU) assay according to the manufacturer's protocol. SW1353 and C28/I2 cells were seeded in 96-well plates at a density of 2 × 10^3^ cells/well for 24 h and then treated and transfected as above. The cells were treated with 50 *μ*M EdU at 37°C for another 2 h, and cell nuclei were stained with DAPI at room temperature for 15 min. Images were obtained by using an inverted fluorescent microscope.

### 2.6. Cell Apoptosis

According to the instructions of the manufacturer, Annexin V-APC/7-AAD kit (BD Pharmingen, San Diego, CA, USA) was used to detect cell apoptosis. SW1353 and C28/I2 cells were treated as above. Subsequently, cells from different treatment groups were resuspended in 500 *μ*L of 1 × binding buffer, stained with Annexin V-APC (5 *μ*L) and 7-AAD (5 *μ*L) for 15 min in the dark, respectively, and then examined using a flow cytometer (Beckman Coulter, CA, USA).

### 2.7. Enzyme-Linked Immunosorbent Assay (ELISA)

The supernatant from each group was collected, and the concentration of IFN-*γ*, TNF-*α*, IL-6, MMP-3, MMP-13, and PGE2 was measured with the appropriate ELISA kit (R&D Systems, Minneapolis, MN USA) according to the manufacturer's instructions.

### 2.8. NO Measurement

Griess reaction method was used to detect NO concentrations in the supernatant according to the manufacturer's instructions. The supernatant was collected from SW1353 and C28/I2 cells which were treated and transfected as mentioned above. An equal volume of cell suspension and Griess reagent was mixed and then incubated for 10 min at room temperature. Absorbance at a wavelength of 550 nm was measured and recorded with a microplate reader (Model 680; Bio-Rad, Hercules, CA, USA).

### 2.9. RT-PCR

Total intracellular RNA from SW1353 and C28/I2 cell was obtained using the TRIzol® Plus RNA Purification Kit (Invitrogen, Carlsbad, CA, USA) according to the manufacturer's instructions. RNA was reverse transcribed to cDNA by using the ThermoScript RT-PCR system (Invitrogen). RT-PCR was performed using SYBR Green PCR Master Mix (Applied Biosystems, Foster City, CA, USA) and the ABI prism 7900HT sequence detection system (Applied Biosystems). GAPDH was used as an internal control. Gene expression was calculated by the 2-*^ΔΔ^*Ct method.

### 2.10. Western Blot

Total protein from SW1353 and C28/I2 cell was obtained using radio-immunoprecipitation assay lysis buffer. Protein concentration was quantified using a BCA Protein Assay kit (Beyotime Biotechnology, Shanghai, China). 30 *μ*g of proteins was separated using 10% SDS-PAGE and then transferred onto the PVDF membranes. The membranes were incubated with primary antibodies overnight at 4°C and sequentially incubated with the corresponding secondary antibodies conjugated by horseradish peroxidase (HRP) for 1 h at room temperature. GAPDH was used as a control. The following primary antibodies were used in this study: rabbit polyclonal to LMX1B antibody (ab139736, 1.5 *μ*g/ml), rabbit monoclonal to NLRP3 antibody (ab263899, 1 : 1000) rabbit monoclonal to p-P65 (ab76302, 1 : 1000), rabbit monoclonal to NF-*κ*B p65 (ab32536, 1 : 5000), and rabbit polyclonal to GAPDH (ab9485, 1 : 2500).

### 2.11. Lentivirus Vector Construction

For the following experiments, recombinant lentivirus vectors pCDH-CMV-MCS-EF1-puro expressing LMX1B siRNA and siRNA control were obtained from Genechem (Shanghai, China).

### 2.12. OA Model in Rats and Animal Treatment

9-10-week-old male Sprague-Dawley rats (270–285 g) were purchased from the Animal Center of Chinese Academy of Sciences (Shanghai, China) and housed with free access to food and water under a 12 h light/dark cycle at room temperature. An animal study in this study was approved by the Animal Care and Use Committee of Central Hospital Affiliated to Shenyang Medical Collage. 28 rats were randomly distributed into four groups: a sham control group (control), an osteoarthritis group (OA), an osteoarthritis treated with control siRNA group (siRNA), and an osteoarthritis treated with LMX1B siRNA group (LMX1B siRNA). Rat knee OA model was established by anterior cruciate ligament transection (ACLT) as described previously [24]. Briefly, the anesthetized rats were made with a parapatellar skin incision on the medial side of the right knee joint; the patella was dislocated before the ACL was transected. Rats in the control group received a sham operation, incision in the joint without ACLT. One week after the surgery, rats were intra-articular injected with LMX1B siRNA lentivirus vector (1 × 10^9^ PFU) or control siRNA lentivirus vector (1 × 10^9^ PFU) 2 times per week. After six weeks of treatment, the rats were sacrificed and knee samples were obtained.

### 2.13. Histological Analysis

Knee samples were fixed with 4% paraformaldehyde, embedded into paraffin, and then cut into 5 *μ*m sections using a rotary microtome. The samples were stained with hematoxylin-eosin (HE) and Safranin O and Fast Green.

### 2.14. Statistical Analysis

Data in the present study were presented as mean ± SD from three independent experiments. Statistical analysis was carried out using the GraphPad Prism 5 software (GraphPad Software, Inc., La Jolla, CA, USA) and Statistical Product and Service Solutions (SPSS) 17.0 (SPSS Inc., Chicago, IL, USA). Differences between groups were analyzed using one-way ANOVA with a subsequent post hoc Tukey's test. *P* < 0.05 was considered statistically significant.

## 3. Results

### 3.1. Expression of LMX1B Was Upregulated in Cartilage Tissue of OA Patients and IL-1*β*-Induced Chondrocytes

**Figure 1:**
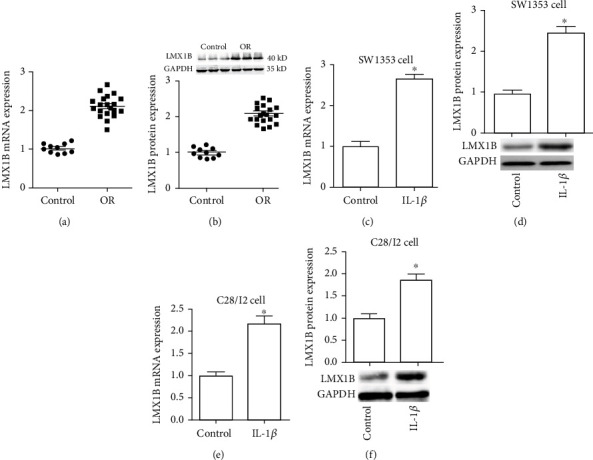
LMX1B was upregulated in OA cartilage tissue and IL-1*β*-induced chondrocytes. (a, c, and e) qRT-PCR analysis for LMX1B mRNA expression in cartilage tissue (a), SW1353 cells (c), and C28/I2 cells (e). (b, d, and f) Western blot analysis for LMX1B protein expression in cartilage tissue (b), SW1353 cells (d), and C28/I2 cells (f). SW1353 and C28/I2 cells were treated with 10 ng/ml IL-1*β* for 24 h. ^∗^*P* < 0.05. 20 OA patients and 10 normal subjects were used in this study; moreover, all experiments were repeated three times in SW1353 and C28/I2 cells.

For investigating the function of LMX1B in OA pathogenesis, we first detected the expression of LMX1B in cartilage tissues of 20 OA patients and 10 normal subjects. Results from the qRT-PCR and western blot assay suggested that LMX1B mRNA and protein levels were significantly increased in OA cartilage tissues against normal control (Figures 1(a) and 1(b)). In the present study, we used 10 ng/ml of IL-1*β* to simulate the OA environment in human chondrosarcoma cell line SW1353 and C28/I2 cells. Moreover, we determined the expression of LMX1B in IL-1*β*-induced SW1353 and C28/I2 cells. qRT-PCR analyses and western blot assay revealed that the mRNA and protein levels of LMX1B were already more expressed in IL-1*β*-induced SW1353 (Figures 1(c) and 1(d)) and C28/I2 (Figures 1(e) and 1(f)) cells in comparison to the control group. Altogether, the above results indicated that LMX1B was increased in OA tissue and cells, and its dysregulate may be involved in OA pathogenesis.

### 3.2. LMX1B Silence Promoted Cell Growth and Suppressed Cell Apoptosis in SW1353 and C28/I2 Cells

**Figure 2:**
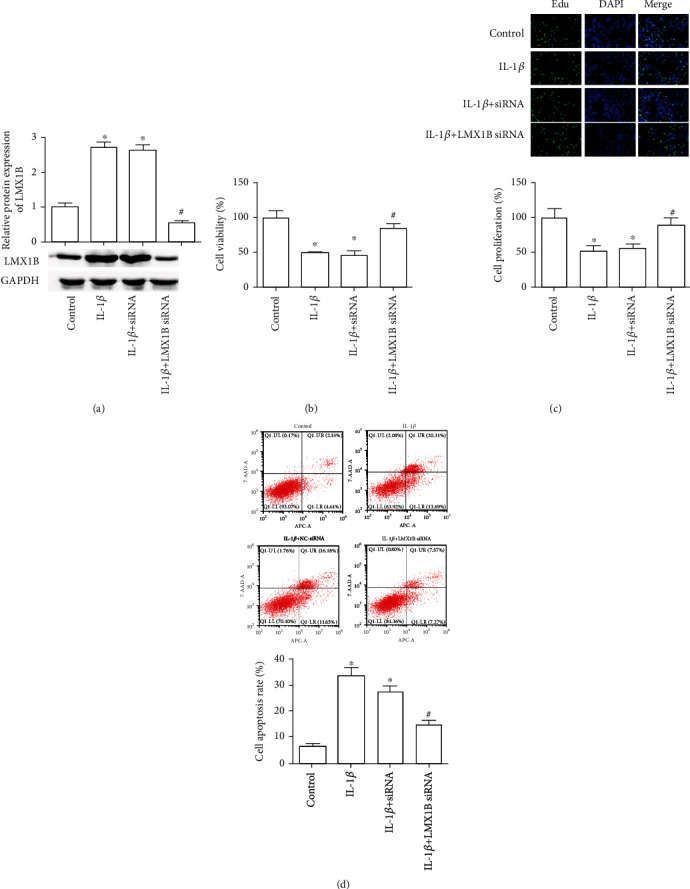
LMX1B knockdown increased IL-1*β*-induced SW1353 cell growth and suppressed its apoptosis. (a) Western blot analysis for LMX1B protein expression; (b) CCK-8 analysis for SW1353 cell survival; (c) 5-ethynyl-2′-deoxyuridine (EdU) assay was used to determine SW1353 cell proliferation; (d) SW1353 cell apoptosis was detected by using flow cytometer analysis. SW1353 cells were transfected with control siRNA or LMX1B siRNA for 24 h by using Lipofectamine 2000 reagent and then treated with 10 ng/ml IL-1*β* for 24 h. ^∗^*P* < 0.05, compared with the control group; ^#^*P* < 0.05, compared with the IL-1*β* group. All experiments were repeated three times.

**Figure 3:**
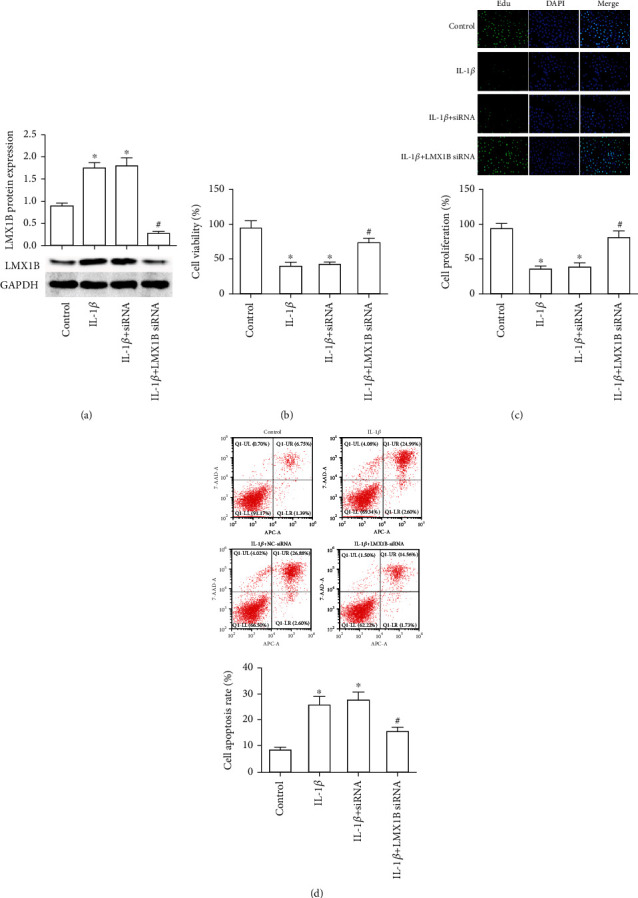
LMX1B knockdown increased IL-1*β*-induced C28/I2 cell growth and suppressed its apoptosis. (a) Western blot analysis for LMX1B protein expression; (b) CCK-8 analysis for C28/I2 cell survival; (c) 5-ethynyl-2′-deoxyuridine (EdU) assay was used to determine C28/I2 cell proliferation; (d) C28/I2 cell apoptosis was detected by using flow cytometer analysis. C28/I2 cells were transfected with control siRNA or LMX1B siRNA for 24 h by using Lipofectamine 2000 reagent and then treated with 10 ng/ml IL-1*β* for 24 h. ^∗^*P* < 0.05, compared with the control group; ^#^*P* < 0.05, compared with the IL-1*β* group. All experiments were repeated three times.

We next sought to investigate whether LMX1B silence mediated SW1353 and C28/I2 cell survival, proliferation, and apoptosis. LMX1B siRNA transfection significantly inhibited LMX1B expression both in SW1353 (Figure 2(a), *P* < 0.05) and C28/I2 (Figure 3(a), *P* < 0.05) cells compared with the IL-1*β* group. The results of the CCK-8 assay suggested that IL-1*β* treatment markedly suppressed SW1353 and C28/I2 cell survival, whereas LMX1B knockdown encouraged cell survival in IL-1*β*-exposed SW1353 (Figure 2(b), *P* < 0.05) and C28/I2 (Figure 3(b), *P* < 0.05) cells. Results from the EdU assay indicated that LMX1B knockdown significantly promoted SW1353 and C28/I2 cell proliferation when compared with the IL-1*β* group (Figures 2(c) and 3(c), *P* < 0.05). Moreover, IL-1*β* treatment resulted in cell apoptosis increased to 34% and 27.58% in SW1353 and C28/I2 cells, respectively; moreover, LMX1B knockdown reversed the effects of IL-1*β* on SW1353 and C28/I2 cell apoptosis (Figures 2(d) and 3(d), *P* < 0.05); cell apoptosis rate in the LMX1B knockdown group was 14.8% and 16.29%. These results imply a regulatory role of LMX1B depletion on IL-1*β*-induced SW1353 and C28/I2 cell survival, proliferation, and apoptosis.

### 3.3. LMX1B Is Essential for the Production of Inflammatory Cytokines in IL-1*β*-Induced SW1353 and C28/I2 Cells

**Figure 4:**
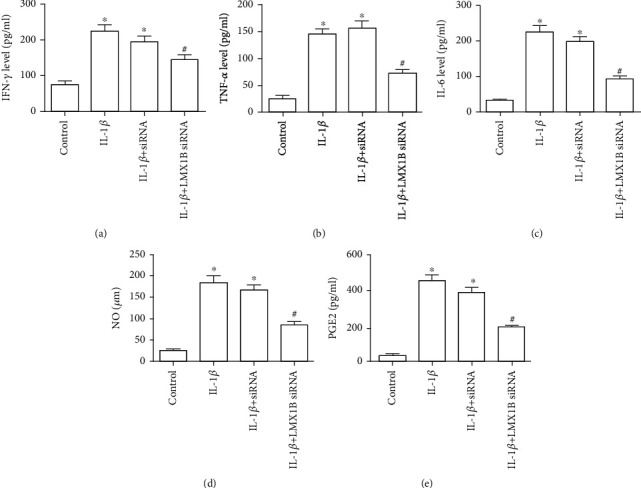
LMX1B depletion suppressed IL-1*β*-induced inflammatory cytokines secretion in SW1353 cells. ELISA kits were used to determine the expression of IFN-*γ* (a), TNF-*α* (b), IL-6 (c), and PGE2 (d); (e) NO concentration was determined by the Griess reaction. SW1353 cells were transfected with control siRNA or LMX1B siRNA for 24 h by using Lipofectamine 2000 reagent and then treated with 10 ng/ml IL-1*β* for 24 h. ^∗^*P* < 0.05, compared with the control group; ^#^*P* < 0.05, compared with the IL-1*β* group. All experiments were repeated three times.

**Figure 5:**
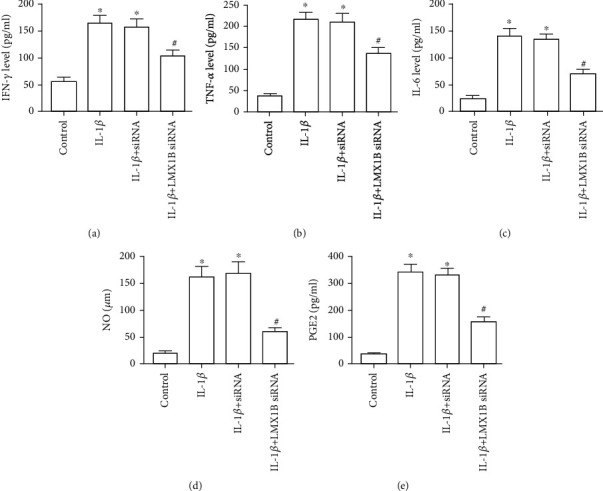
LMX1B depletion suppressed IL-1*β*-induced inflammatory cytokines secretion in C28/I2 cells. ELISA kits were used to determine the expression of IFN-*γ* (a), TNF-*α* (b), IL-6 (c), and PGE2 (d); (e) NO concentration was determined by the Griess reaction. C28/I2 cells were transfected with control siRNA or LMX1B siRNA for 24 h by using Lipofectamine 2000 reagent and then treated with 10 ng/ml IL-1*β* for 24 h. ^∗^*P* < 0.05, compared with the control group; ^#^*P* < 0.05, compared with the IL-1*β* group. All experiments were repeated three times.

Next, to validate the effects of LMX1B knockdown on the inflammation response in IL-1*β*-induced chondrocyte, the expression of inflammatory cytokines IFN-*γ*, TNF-*α*, IL-6, PGE2, and NO was analyzed by the appropriate ELISA kit according to the manufacturer's instructions. IFN-*γ*, TNF-*α*, IL-6, PGE2, and NO levels were already significantly increased after IL-1*β* treatment compared to the control group (Figures 4 and 5, *P* < 0.05). Additionally, at IL-1*β*+control siRNA group, IFN-*γ*, TNF-*α*, IL-6, PGE2, and NO were all almost unchanged compared to the IL-1*β*-treated group (Figures 4 and 5, *P* > 0.05), and importantly, these inflammatory cytokines were considerably downregulated compared to the IL-1*β*-treated group and IL-1*β*+control siRNA group (Figures 4 and 5, *P* < 0.05).

### 3.4. LMX1B Depletion Plays Important Role in IL-1*β*-Induced MMP3 and MMP13 Expressions in SW1353 and C28/I2 Cells

**Figure 6:**
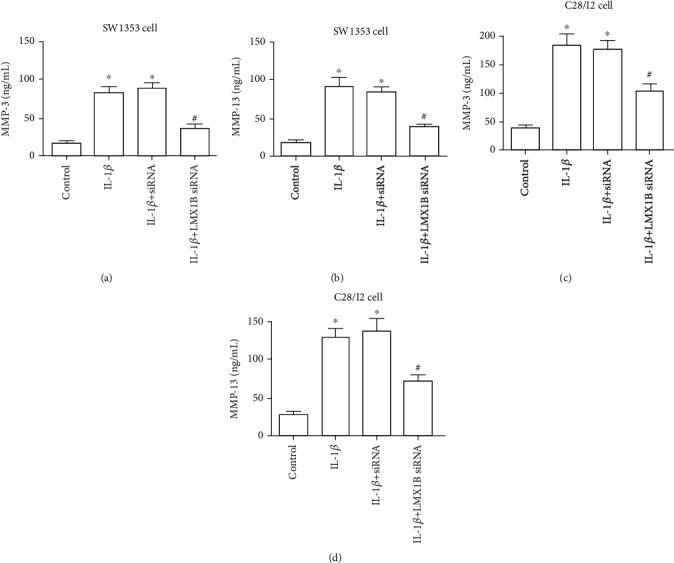
LMX1B depletion suppressed IL-1*β*-induced MMP3 and MMP13 expression. ELISA kits were used to determine the expression of MMP3 (a and c) and MMP13 (b and d). SW1353 and C28/I2 cells were transfected with control siRNA or LMX1B siRNA for 24 h by using Lipofectamine 2000 reagent and then treated with 10 ng/ml IL-1*β* for 24 h. ^∗^*P* < 0.05, compared with the control group; ^#^*P* < 0.05, compared with the IL-1*β* group. All experiments were repeated three times.

In order to assess whether the LMX1B depletion mediated MMP3 and MMP13 expressions in IL-1*β*-induced SW1353 and C28/I2 cells, ELISA assay was carried out. In fact, stimulation with IL-1*β* significantly increased the release of MMP3 and MMP13 into the culture medium both in SW1353 (Figures 6(a) and 6(b), *P* < 0.05) and C28/I2 cells (Figures 6(c) and 6(d), *P* < 0.05). Additionally, MMP3 and MMP13 expressions were significantly reduced after LMX1B siRNA transfection as compared with the IL-1*β* group (Figure 6).

### 3.5. LMX1B Knockdown Inhibits NF-*κ*B Activation and Attenuates NLRP3 Expression

**Figure 7:**
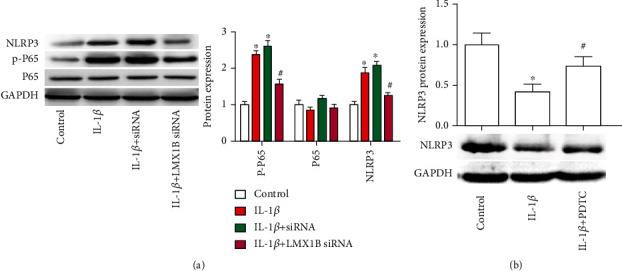
LMX1B depletion suppressed IL-1*β*-induced NF-*κ*B and NLRP3 activation. Western blot was used to determine the protein expression of p-P65, P65 (a), and NLRP3 (b). SW1353 cells were transfected with control siRNA or LMX1B siRNA for 24 h by using Lipofectamine 2000 reagent and then treated with 10 ng/ml IL-1*β* for 24 h, or SW1353 cells were treated with 10 ng/ml IL-1*β* and PDTC for 24 h. ^∗^*P* < 0.05, compared with the control group; ^#^*P* < 0.05, compared with the IL-1*β* group. All experiments were repeated three times.

To understand the regulatory role of LMX1B, NF-*κ*B, and NLRP3 signal pathway has been investigated. As shown in Figure 7(a), IL-1*β* proinflammatory stimulus enhanced NF-*κ*B signal pathway activation through promoting p-P65 expression, whereas total P65 expression was unchanged. Moreover, IL-1*β* treatment significantly increased NLRP3 expression, compared with the control group (*P* < 0.05). Additionally, expression of p-P65 and NLRP3 in the IL-1*β*+LMX1B siRNA transfection group markedly declined, compared with the IL-1*β* group (Figure 7(a), *P* < 0.05). Additionally, we observed that the NF-*κ*B activation and NLRP3 expression showed a tight positive correlation in SW1353 cells treated with IL-1*β*. Results from western blot assay showed that the inhibitor of NF-*κ*B signal pathway reversed the effects of IL-1*β* on the expression of NLRP3 (Figure 7(b)). The above results indicated that LMX1B silence inhibited NLRP3 expression which may be through inhibiting NF-*κ*B activation.

### 3.6. NLRP3 Overexpression or NF-*κ*B Activation Restrained the Effects of LMX1B Silence on SW1353 Cell Biology

**Figure 8:**
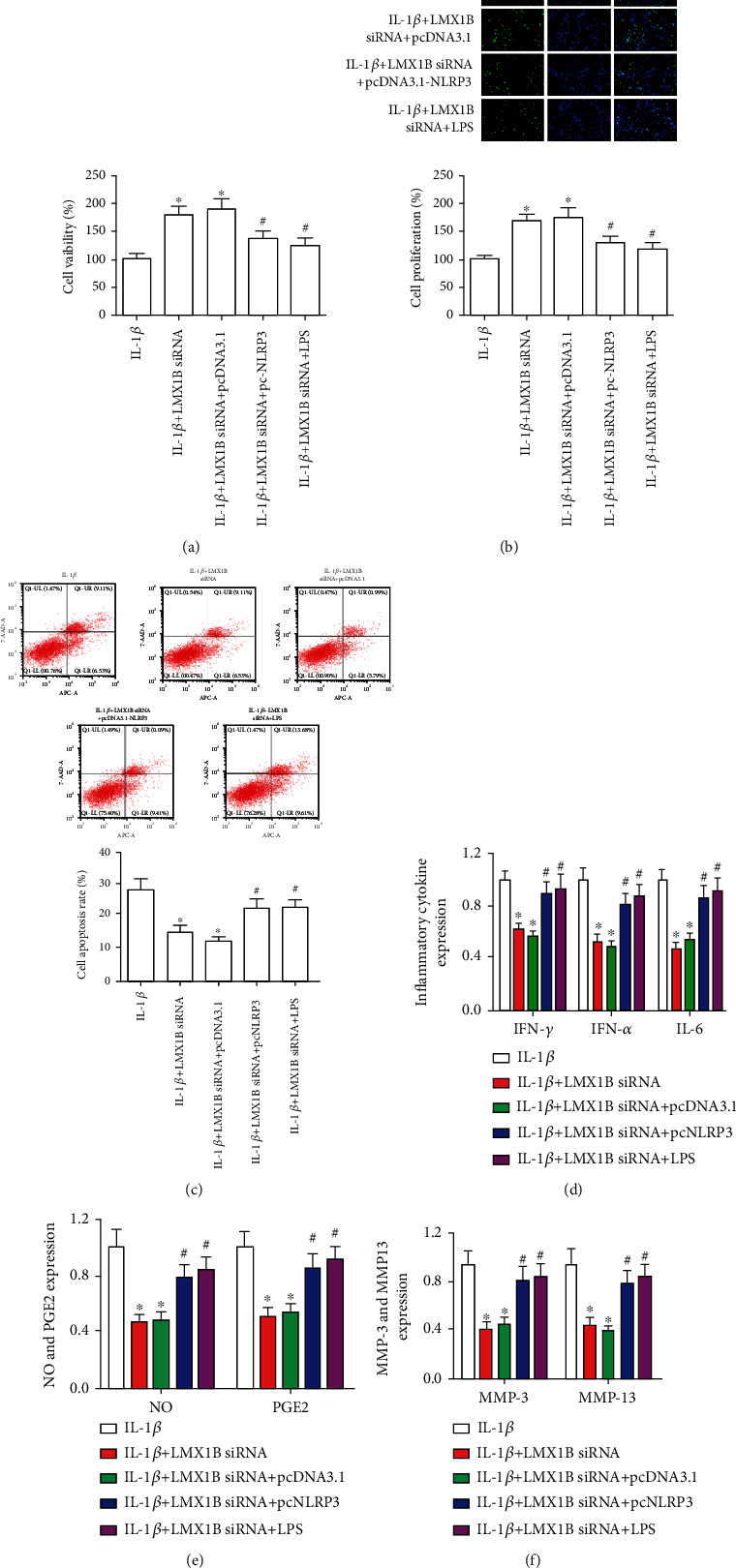
NLRP3 overexpression or NF-*κ*B activation reversed the effects of LMX1B silence on SW1353 cell biology. (a) CCK-8 analysis for SW1353 cell survival; (b) 5-ethynyl-2′-deoxyuridine (EdU) assay was used to determine SW1353 cell proliferation; (c) SW1353 cell apoptosis was detected by using flow cytometer analysis. (d) ELISA kits were used to determine the expression of IFN-*γ*, TNF-*α*, IL-6, and PGE2; (e) NO and PGE2 concentration was determined by the Griess reaction and ELISA kit, respectively; (f) ELISA kits were used to determine the expression of MMP3 and MMP13. SW1353 cells were transfected with LMX1B siRNA for 24 h by using Lipofectamine 2000 reagent and then treated with 10 ng/ml IL-1*β* for 24 h or SW1353 cells were cotransfected with LMX1B siRNA and pcDNA3.1 control vector or LMX1B siRNA and pcDNA3.1-NLRP3 vector for 24 h and then treated with 10 ng/ml IL-1*β* for 24 h, or SW1353 cells were treated with 10 ng/ml IL-1*β* and LPS (400 *μ*g/ml) for 24 h. ^∗^*P* < 0.05, compared with the IL-1*β* group; ^#^*P* < 0.05, compared with the IL-1*β*+LMX1B siRNA group. All experiments were repeated three times.

To investigate the effects of NF-*κ*B and NLRP3 signal pathway on the action of LMX1B silence in IL-1*β*-induced SW1353 cells, we used NF-*κ*B activator LPS (400 *μ*g/ml) and engineered NLRP3 overexpression plasmids for restoring the expression of p-P65 and NLRP3. As shown in Figures 8(a) and 8(b), NF-*κ*B activation and LMX1B overexpression significantly suppressed cell survival and proliferation compared with the LMX1B knockdown group (*P* < 0.05). We also examined the influence of LPS and LMX1B overexpression vector on cell apoptosis, inflammatory cytokines, and MMP3 and MMP13 expression in LMX1B silence and IL-1*β*-induced SW1353 cells. Cell apoptosis, IFN-*γ*, TNF-*α*, IL-6, PGE2, NO, MMP3, and MMP13 expressions were augmented significantly after exposure to LPS and transfecting with LMX1B overexpression vector, compared with LMX1B knockdown group (*P* < 0.05). These results indicated that LMX1B silence plays effective roles in cell apoptosis and inflammatory response dependent on NF-*κ*B and NLRP3 signal pathway.

### 3.7. LMX1B Depletion Suppressed Pathogenesis of Osteoarthritis in Sprague-Dawley Rats

**Figure 9:**
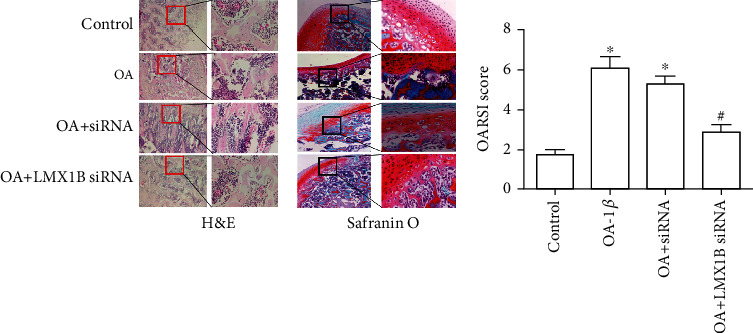
LMX1B depletion suppressed pathogenesis of osteoarthritis in Sprague-Dawley rats. Representative images of HE and Safranin O staining, OARSI grade in control, OA, control siRNA, and LMX1B siRNA group. ^∗^*P* < 0.05, compared with the control group; ^#^*P* < 0.05, compared with the OA group. In the study, 7 rats were used in each group.

**Figure 10:**
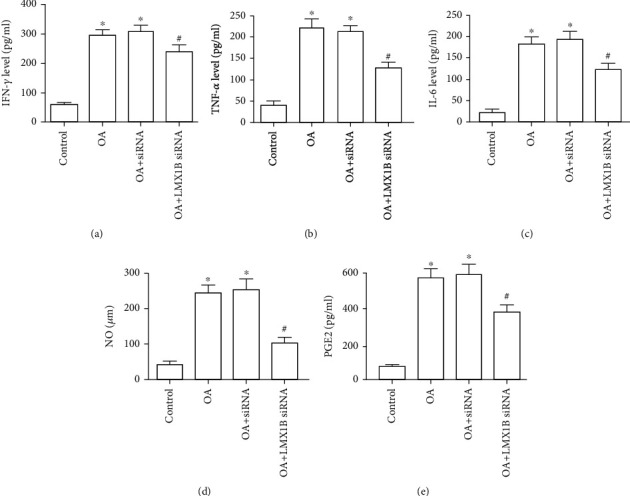
LMX1B depletion suppressed inflammatory cytokines secretion in OA rats. ELISA kits were used to determine the expression of IFN-*γ* (a), TNF-*α* (b), IL-6 (c), and PGE2 (e); (d) NO concentration was determined by the Griess reaction. ^∗^*P* < 0.05, compared with the control group; ^#^*P* < 0.05, compared with the OA group. In the study, 7 rats were used in each group.

We next assessed whether LMX1B depletion plays roles in the regulation of OA cartilage destruction. Lentiviruses with LMX1B siRNA were injected into the knee joints of OA rats. Hematoxylin and eosin (HE) and Safranin O staining were performed. In the results as shown in Figure 9, compared with the control group, OA group rats exhibited osteoarthritis characteristics with severely degraded cartilage and clear hypocellularity. Moreover, we observed that LMX1B depletion significantly alleviated symptoms of OA (Figure 9). Moreover, IFN-*γ*, TNF-*α*, IL-6, PGE2, and NO levels were already significantly increased in OA rats, and these inflammatory cytokines were considerably downregulated after LMX1B depletion (Figures 10(a)–10(e), *P* < 0.05). These data indicated that LMX1B depletion had protective effects *in vivo*.

## 4. Discussion

Osteoarthritis (OA) is a highly prevalent chronic disorder of joints featuring degeneration of articular cartilage. Up to now, treatment of osteoarthritis remains a difficult problem, and although physical therapy and pain medications are used, there have been limited effects and significant side effects [1, 25]. The present study firstly provided evidence that LMX1B knockdown plays effective inhibition roles in the pathogenesis of OA. Firstly, we found that LMX1B expression presented a significant elevation in OA patients' tissues and IL-1*β*-induced human chondrocytes. Secondly, we observed that LMX1B silence promoted cell survival and proliferation and suppressed cell apoptosis and inflammatory response in IL-1*β*-induced human osteoarthritis chondrocytes. Thirdly, we demonstrated that NF-*κ*B/NLRP3 signal pathway plays an irreplaceable role in the action of LMX1B depletion in IL-1*β*-induced chondrocytes. Finally, we used OA model in Sprague-Dawley rats to confirm the protective effects of LMX1B silence against OA. Based on our data, we suggested that LMX1B might play an important role in the progression of OA.

LIM homeodomain LMX1B was composed of two cysteine-enriched, zinc-binding NH_2_-terminal LIM domains, including a COOH-terminal glutamine-rich domain and a homeodomain consisting of 60 amino acids [26, 27]. Evidence suggested that LMX1B is widely expressed in types of human tissues, including testis, thyroid, pancreatic islets, ocular, and skeletal muscle [15, 28, 29]. LMX1B has been shown associated with cell proliferation, migration, apoptosis, and inflammatory cytokine production [30–32]. As a transcription factor, LMX1B could regulate multiple pathways and cell physiologies via mediating associated target genes [11]. Microarray analysis from the previous reports suggested that LMX1B upregulated NF-kappaB target genes, including IL-6 and IL-8 expressions in tetracycline-inducible HeLa cells [30]. It is now well established that inflammatory cytokine IL-1*β* is involved in the pathogenesis of OA through mediating matrix metalloproteinases (MMPs) and inflammatory cytokines expression [33, 34]. Also, evidence indicated that MMP members MMP-3 and MMP-13 are the most important enzymes in OA [35]. In the present study, we demonstrated that LMX1B plays important roles in IL-1*β*-induced inflammatory cytokines and MMP-3 and MMP-13 expressions.

We next determined the effects of LMX1B on the expression of NF-*κ*B-p65, and the results from WB suggested that LMX1B knockdown significantly suppressed IL-1*β*-induced NF-*κ*B activation through inhibiting p-p65 expression. Emerging studies have revealed that NF-*κ*B proteins are a member of the transcription factor family which is stimulated by chemokine, extracellular matrix (ECM) degradation products, stress-related factors, and proinflammatory cytokines [36]. A recent study also suggested that the expression of the NLRP3 inflammasome was accompanied by the activation of Toll-like receptors (TLRs) and NF-*κ*B [37]. In the present study, we confirmed that NF-*κ*B inhibitor suppressed NLRP3 expression. In addition, evidence suggested that NLRP3 contributed to inflammatory cytokine production [38]. Moreover, recent studies indicated that NLRP3 was a potentially novel biomarker of OA and might play essential roles in the pathogenesis of OA [39, 40]. In the current study, we observed that LMX1B depletion suppressed NF-*κ*B-p65 activation and NLRP3 expression, and NF-*κ*B-p65 activation and NLRP3 overexpression both reversed the effects of LMX1B knockdown on chondrocytes apoptosis and inflammation.

## 5. Conclusion

Collectively, the present study provided the first evidence that LMX1B knockdown exerts protective activity in IL-1*β*-induced human chondrocytes and OA model. Also, the possible mechanisms of LMX1B knockdown in IL-1*β*-induced chondrocytes might involve the inactivation of NF-*κ*B and the inhibition of NLRP3. In summary, LMX1B knockdown may be effective in the treatment of OA.

## Data Availability

The datasets used and analyzed during the current study are available from the corresponding author on reasonable request.
